# Oxygen-Vacancy-Rich TiO_2_ Nanosheets with High Stability for Efficient Photocatalytic Cr(VI) Reduction

**DOI:** 10.3390/nano16130832

**Published:** 2026-07-07

**Authors:** Yingjie Jiang, Xiaoli Jia, Li Fang, Qin Zhang, Ruiting Li, Bingqian Zhao, Jiancong Liu, Yaorui Li

**Affiliations:** 1 Key Laboratory of Functional Inorganic Material Chemistry, Ministry of Education of the People’s Republic of China, Heilongjiang University, Harbin 150080, China; jiangyingjie0919@163.com (Y.J.); m18535474685@163.com (X.J.); fangli02242000@163.com (L.F.); zhangaqin@163.com (Q.Z.); 19836405899@163.com (R.L.); 15090632762@163.com (B.Z.); 2 Heilongjiang Provincial Key Laboratory of Nuclear Chemical Engineering and Radiochemistry, College of Nuclear Science and Technology, Harbin Engineering University, Harbin 150001, China

**Keywords:** TiO_2_ nanosheets, defect engineering, high stability, hydrogen reduction, Cr(VI) photoreduction, photocatalysis

## Abstract

Defect engineering of anatase TiO_2_ nanosheets by hydrogen reduction is a compelling strategy to boost visible light photocatalytic Cr(VI) reduction, a process of vital importance for detoxifying highly toxic and carcinogenic Cr(VI) pollutants. However, the necessary high-temperature hydrogen treatment invariably induces morphological collapse, negating the structural merits of the two-dimensional nanosheets. Herein, we propose an ethylenediamine reflux protection strategy combined with hydrogen reduction to fabricate defect-rich TiO_2_ nanosheets (EN-TiO_2−x_-NS) that preserve the original morphology. The resulting EN-TiO_2−x_-NS retained the square nanosheet structure and (001) facets, while Ti^3+^ and oxygen vacancies were successfully introduced. The bandgap narrowed from 2.95 to 2.55 eV, leading to enhanced visible light absorption and charge separation efficiency. For photocatalytic Cr(VI) reduction under visible light, EN-TiO_2−x_-NS achieved a removal rate of 97.3% within 20 min, with a rate constant 1.93 times higher than that of pristine TiO_2_ nanosheets and 3.17 times higher than that of the directly hydrogenated sample. The catalyst also exhibited excellent cycling stability. This work demonstrates a synergistic strategy combining morphology preservation and defect engineering, providing a new approach for designing high-performance TiO_2_-based photocatalysts.

## 1. Introduction

The rapid expansion of industries such as tanning, electroplating, and metallurgy has led to an ever-increasing annual discharge of chromium-containing wastewater, and the resulting ecological pollution problems have drawn growing attention [1,2,3]. Hexavalent chromium (Cr(VI)) in water, due to its extremely high carcinogenicity, good solubility, and strong mobility, has been listed as a priority pollutant by the U.S. Environmental Protection Agency [4]. In contrast, Cr(III) is harmless to humans and is an essential element [5]; reducing Cr(VI) to a less toxic state is therefore an effective approach for treating chromium-containing wastewater [6,7,8]. Traditional methods for removing Cr(VI) include chemical precipitation [9], filtration [10], and adsorption [11]. Although these methods are effective to some extent, they suffer from drawbacks such as the risk of secondary pollution, high cost, or relatively complicated operation procedures. In this context, photocatalytic technology based on solar energy conversion, which is environmentally friendly, becomes particularly important [12,13]. As a key photocatalytic material, titanium dioxide (TiO_2_) has attracted considerable interest owing to its good chemical stability, resistance to photocorrosion, and low cost [14,15,16,17]. Different morphologies of anatase TiO_2_ correspond to different specific surface areas, surface atomic arrangements, and coordination environments, thereby influencing the ultimate photocatalytic activity [18,19,20,21]. Numerous studies have found that anatase TiO_2_ with a sheet-like structure exhibits excellent surface activity due to its large specific surface area and abundant unsaturated titanium atoms, which play a positive role in enhancing the application prospects of such materials [22,23]. The pioneering work of Yang et al. has demonstrated that using F^-^ as a morphology-controlling agent can successfully synthesize anatase TiO_2_ with a high proportion of highly active sheet-like morphology [23]. Applying nanosheet TiO_2_ to the photocatalytic reduction in Cr(VI) is expected to deliver superior catalytic performance by virtue of its unique structural advantages. However, the relatively large band gap of anatase TiO_2_ nanosheets (ca. 3.2 eV) leads to insufficient utilization of visible light, while the rapid recombination of photogenerated electron-hole pairs severely restricts the overall photocatalytic efficiency [24,25,26,27].

To address these issues, while preserving the two-dimensional structural features, it is urgently necessary to develop an effective strategy that can extend the visible light response range of TiO_2_ and enhance the carrier separation efficiency [28]. Introducing oxygen vacancies into TiO_2_ is an effective strategy to enhance visible light absorption and improve photocatalytic activity. To this end, various methods have been developed, each with its own merits and limitations (Table S1). Metal element doping offers a convenient and effective pathway for generating oxygen vacancies, although the resulting defect concentration may be limited and foreign elements may also be introduced [29,30]. Heterojunction induction can promote oxygen vacancy formation through interfacial effects, yet the induced defects are typically confined to the interfacial region [31]. Chemical reduction methods, such as NaBH_4_ treatment, offer mild operating conditions, but generally generate only a low concentration of surface oxygen vacancies [32]. High-temperature hydrogen reduction is highly effective in introducing a high concentration of bulk defects; however, the harsh reduction conditions may cause structural collapse and material sintering [33,34]. Therefore, developing a strategy that can introduce sufficient defects while maintaining the structural integrity of the catalyst remains highly desirable. Various morphology protection strategies have been reported, but these approaches tend to be relatively complex and require multiple steps [35]. Therefore, developing a simple strategy that can simultaneously achieve efficient oxygen vacancy introduction and microstructural preservation is of great significance for advancing the practical application of oxygen vacancy engineering in photo-catalytic Cr(VI) reduction.

In this study, anatase TiO_2_ nanosheets with exposed highly active facets were used as the platform to develop a surface defect-rich photocatalyst via a combined strategy of ethylenediamine (EDA) reflux protection and hydrogen reduction. The resulting material (EN-TiO_2−x_-NS) successfully retained its square nanosheet morphology and (001) facets, while Ti^3+^ and oxygen vacancies were effectively introduced, as confirmed by XPS and EPR. The bandgap narrowed from 2.95 eV to 2.55 eV, leading to markedly enhanced visible light absorption and improved charge separation. Under visible light irradiation, the optimized EN-TiO_2−x_-NS achieved a Cr(VI) removal rate of 97.3% within 20 min, with a reaction rate constant 1.93 times and 3.17 times higher than those of pristine TiO_2_ nanosheets and the directly hydrogenated sample, respectively. This work demonstrates an effective approach for introducing defects while preserving the structural advantages of the highly active morphology, offering new insights for the rational design of high-performance TiO_2_-based photocatalysts.

## 2. Materials and Methods

### 2.1. Materials

Tetrabutyl titanate (TBOT, ≥99%) was purchased from Shanghai Aladdin Biochemical Technology Co., Ltd. (Shanghai, China), Ethylenediamine (EDA, 99.5%) was purchased from Shandong Xiya Chemical Co., Ltd. (Heze City, Shandong Province, China), K_2_Cr_2_O_7_ (≥99.5%) was purchased from Shanghai Aladdin Biochemical Technology Co., Ltd. (Shanghai, China). HF solution (40 wt%) was purchased from Tianjin Kemiou Chemical Reagent Co., Ltd. (Tianjin, China). All chemicals and materials were obtained from commercial sources and used without further purification.

### 2.2. Materials Preparation

#### 2.2.1. Synthesis of the TiO_2_-NS

First, 25 mL of tetrabutyl titanate was measured into a 100 mL PTFE-lined stainless-steel autoclave, and 4 mL of HF solution (40 wt%) was slowly added dropwise under magnetic stirring. After stirring until homogeneous, the autoclave was sealed and placed in an oven at 200 °C for 20 h. Once naturally cooled to room temperature, the product was collected by centrifugation, washed successively with deionized water and 0.1 mol/L NaOH solution until neutral, and then dried at 60 °C overnight to obtain TiO_2_-NS.

#### 2.2.2. Synthesis of EN-TiO_2−x_-NS

TiO_2_-NS was first prepared as described above. Then, 1.0 g of the as-prepared TiO_2_-NS was dispersed in a round-bottom flask containing 6 mL of EDA and 120 mL of deionized water. The mixture was refluxed at 85 °C in an oil bath with stirring for 48 h. After naturally cooling to room temperature, the precipitate was collected by centrifugation, washed several times with deionized water and absolute ethanol, and dried at 60 °C overnight. The dried sample was calcined in a muffle furnace at 450 °C for 2 h and subsequently placed in a tube furnace under a hydrogen atmosphere, heated to 500 °C at a heating rate of 1 °C/min, and held at that temperature for 2 h. The resulting sample was denoted as EN-TiO_2−x_-NS.

To investigate the protective effect of the EDA reflux treatment, the TiO_2_-NS sample without ethylenediamine reflux was directly subjected to the same treatment, and the obtained control sample was denoted as TiO_2−x_-NS.

### 2.3. Material Characterizations

Powder X-ray diffraction (XRD) analysis was performed on a Bruker D8 diffractometer (Bruker AXS GmbH, Karlsruhe, Germany) with Cu Kα radiation (λ = 1.5406 Å) operated at 40 kV.

Scanning Electron Microscopy (SEM) was performed using a ZEISS Sigma-500 field-emission scanning electron microscope (SEM, Carl Zeiss Microscopy GmbH, Ober-kochen, Germany) to observe the sample morphology, with a test voltage of 5 kV.

Transmission electron microscopy (TEM) measurements were carried out on a JEM-2100 microscope (TEM, JEOL Ltd., Tokyo, Japan) operated at 200 kV. X-ray photoelectron spectroscopy (XPS) was acquired on a VG ESCALAB MK II instrument (XPS, Thermo Fisher Scientific, Waltham, MA, USA) equipped with an Mg Kα (1253.6 eV) source.

Solid-State Electron Paramagnetic Resonance (EPR) The electron paramagnetic resonance (EPR) spectra of the samples were measured using a Bruker EMX plus spectrometer (Bruker BioSpin Corp., Billerica, MA, USA).

N_2_ adsorption–desorption isotherm analysis: N_2_ adsorption-desorption isotherms were collected using a TriStar II 3020 fully automated analyzer (Micromeritics Instrument Corp., Norcross, GA, USA).

UV-Vis absorption spectroscopy (UV-Vis): The UV-Vis absorption range of the materials was determined using a Shimadzu UV-2550 UV-Vis (Shimadzu Corp., Kyoto, Japan) diffuse reflectance spectrometer.

Scanning Kelvin Probe (SKP) Analysis SKP analysis was performed using a Scottish-manufactured SKP5050 system (KP Technology Ltd., Scotland, UK) in ambient air at atmospheric pressure, with a gold electrode serving as the reference electrode.

### 2.4. Photocatalytic Cr(VI) Reduction Performance Test

Photocatalytic reduction in Cr(VI) was performed using a 300 W xenon lamp (light intensity: 0.8 W/cm^2^, Beijing Perfectlight, PLS-SXE300C, Beijing, China) with the as-prepared photocatalysts. The pH of the solution was adjusted with small amounts of NaOH (0.01 mol/L) and H_2_SO_4_ (0.01 mol/L). In a typical experiment, 20 mg of photocatalyst was dispersed in 100 mL of Cr(VI) solution with an initial concentration of 10 mg/L. Prior to irradiation, the suspension was stirred in the dark for 30 min. At given time intervals, 1.0 mL aliquots were sampled. The Cr(VI) concentration was determined by the diphenyl carbazide colorimetric method. In cycling tests, after each photocatalytic reaction, the catalyst was ultrasonically cleaned in deionized water for 2 h, then washed three times with deionized water and absolute ethanol, and finally dried at 60 °C overnight.

The removal of Cr(VI) is expressed as C_t_/C_0_, where C_0_ and C_t_ represent the initial Cr(VI) concentration and the concentration at time t, respectively.

The Cr(VI) removal efficiency (R, %) was calculated by the following equation:

R = [(C_0_ − C_t_)/C_0_] × 100% (1)

The Cr(VI) adsorption capacity (q, mg·g^−1^) was calculated as:

Q = (C_0_ − C_t_) × V/m (2)

The photocatalytic reduction kinetics (k) were described by the pseudo-first-order kinetic equation:

−In (C_t_/C_0_′) = kt (3)

where C_0_′ is the Cr(VI) concentration after dark adsorption, C_t_ is the Cr(VI) concentration at irradiation time t (min), and k is the pseudo-first-order rate constant (min^−1^).

## 3. Results and Discussion

### 3.1. Characterization of EN-TiO_2−x_-NS

**Figure 1:**
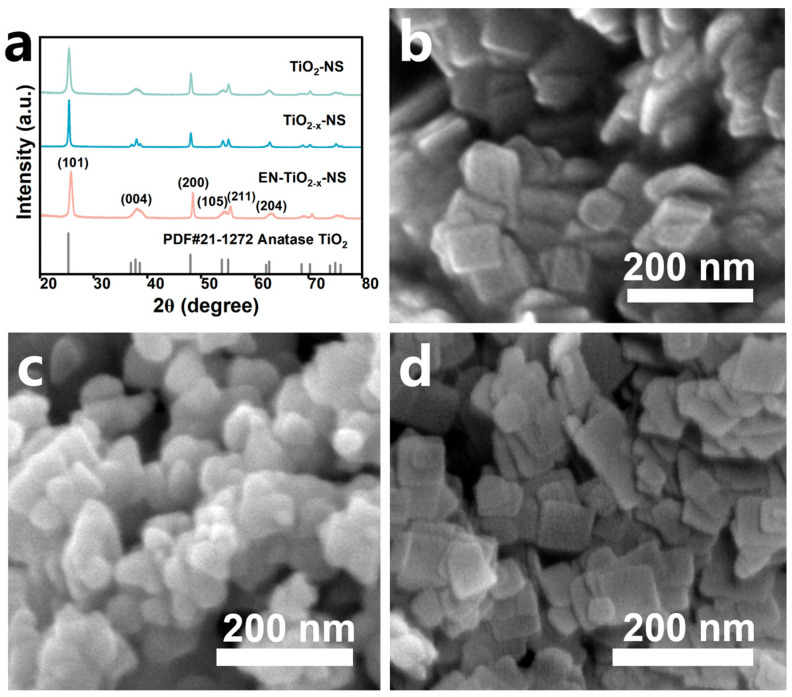
(**a**) XRD patterns of the TiO_2_-NS, TiO_2−x_-NS and EN-TiO_2−x_-NS. (**b**–**d**) SEM image of the TiO_2_-NS, TiO_2−x_-NS and EN-TiO_2−x_-NS.

The crystal structures of the as-prepared samples were characterized by X-ray diffraction (XRD), and the results are shown in Figure 1a. All samples exhibit characteristic diffraction peaks of anatase TiO_2_ (JCPDS No. 21-1272), with no diffraction peaks corresponding to rutile or other impurity phases detected, indicating that the hydrogen reduction treatment did not induce a phase transition. To further analyze the influence of different treatments on the crystal structure, the intensity ratio of the (101) to (200) diffraction peaks, denoted as I_(101)_/I_(200)_, was evaluated. According to literature reports, a lower I_(101)_/I_(200)_ ratio generally indicates a higher exposure proportion of (001) facets [36]. The pristine TiO_2_-NS sample shows an I_(101)_/I_(200)_ ratio of 2.1. For the directly hydrogen-reduced TiO_2−x_-NS sample, this ratio increases to 3.3, presumably because some high-index facets undergo reconstruction or preferential orientation growth driven by thermodynamics, leading to a relative weakening of the (200) diffraction. In contrast, the EN-TiO_2−x_-NS sample, which was pretreated by EDA reflux before hydrogenation, exhibits an I_(101)_/I_(200)_ ratio of 1.8, which is close to that of pristine TiO_2_-NS, indicating that the EDA protection effectively preserved the original facet characteristics during hydrogenation.

The morphology of the samples before and after hydrogenation was characterized by SEM, and the results are shown in Figure 1. As can be seen from Figure 1b, the pristine TiO_2_-NS sample exhibits a typical square nanosheet morphology with well-defined edges, a regular shape. For the TiO_2−x_-NS sample directly subjected to hydrogen reduction without EDA reflux protection (Figure 1c), the morphology changed significantly from square nanosheets to nanoparticles. As shown by the XRD pattern in Figure 1a, the intensity of the (200) diffraction peak near 2θ = 20° for TiO_2−x_-NS is obviously reduced, indicating that under the high-temperature conditions, both the morphology and the surface crystal facets underwent changes. In contrast, the EN-TiO_2−x_-NS sample obtained by EDA reflux followed by hydrogen reduction (Figure 1d) largely retains the original morphology, remaining as square nanosheets with a uniform size.

**Figure 2:**
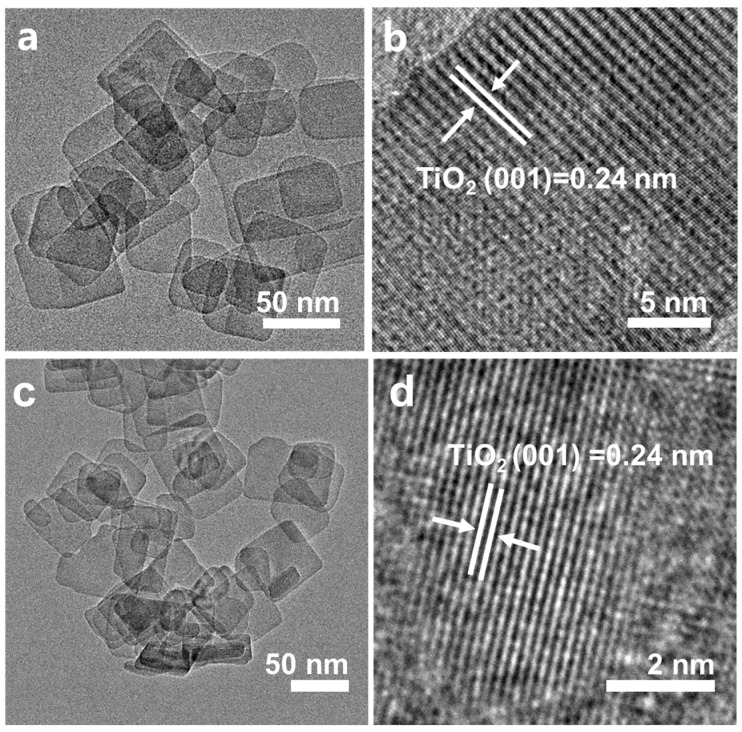
TEM and HRTEM images of (**a**,**b**) TiO_2_-NS, (**c**,**d**) EN-TiO_2−x_-NS.

TEM and HRTEM were further employed to investigate the microstructural details [37]. The HRTEM image of pristine TiO_2_-NS shows clear lattice fringes with a spacing of 0.24 nm (Figure 2a,b), which corresponds to the (001) plane of anatase TiO_2_, indicating that the exposed facets are mainly the highly active (001) facets [38,39]. For the EN-TiO_2−x_-NS sample, the HRTEM image reveals clear lattice fringes with a spacing of 0.24 nm (Figure 2c,d), assigned to the (001) plane of anatase TiO_2_, confirming the preservation of the highly active (001) facets. In contrast, the TiO_2−x_-NS sample exhibits lattice fringes with a spacing of 0.35 nm, corresponding to the (101) plane of anatase TiO_2_ (Figure S1), indicating that the (001) facets were largely lost during unprotected hydrogen reduction and replaced by the thermodynamically stable (101) facets. The specific surface areas and BJH pore volumes of the three samples are summarized in Table S2 and Figures S2–S4. Pristine TiO_2_-NS exhibits a surface area of 98 m^2^/g and a pore volume of 0.27 cm^3^/g. After direct high-temperature hydrogen reduction, TiO_2−x_-NS shows decreases in both surface area (90 m^2^/g) and pore volume (0.22 cm^3^/g), consistent with nanosheet collapse and agglomeration. In contrast, EN-TiO_2−x_-NS, prepared via EDA reflux protection followed by hydrogen reduction, retains a surface area of 96 m^2^/g and a pore volume of 0.30 cm^3^/g, both close to those of pristine TiO_2_-NS, indicating that the EDA pretreatment effectively inhibits structural damage during hydrogenation. This structural preservation enables the TiO_2_ to retain its original morphology and highly active facets while successfully introducing oxygen vacancies and Ti^3+^ defects, thereby broadening the visible light absorption range and improving the overall photocatalytic efficiency [40,41].

The XRD and SEM/TEM results clearly demonstrate that the EDA treatment effectively preserves the nanosheet morphology during hydrogenation. To understand the origin of this protective effect, the interaction between EDA and the TiO_2_ surface was investigated by FTIR and XPS. The corresponding data are provided in the Supporting Information (Figures S5–S7). First, the FTIR spectrum (Figure S5) of the EDA-treated intermediate before calcination exhibits characteristic N–H, C–H, and C–N vibrational bands, confirming the presence of EDA on the TiO_2_ surface [42,43]. After reduction, however, these absorption bands completely disappear, indicating the removal of the organic species. XPS N 1s analysis further confirms the chemical adsorption of EDA (Figure S6). No nitrogen signal is detected in the XPS survey spectrum of the final product (Figure S7). These results indicate that EDA enables defect introduction without compromising the morphology, while being cleanly removed in the process.

**Figure 3:**
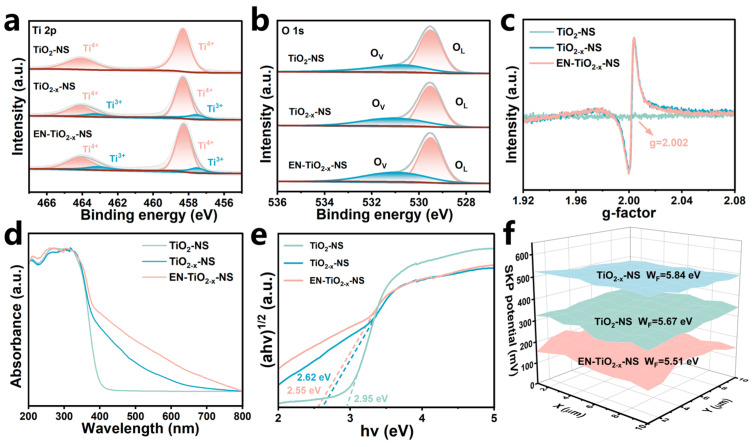
(**a**) Ti 2p, (**b**) O 1s high resolution XPS spectra of TiO_2_-NS, TiO_2−x_-NS and EN-TiO_2−x_-NS. (**c**) EPR spectrum of TiO_2_-NS, TiO_2−x_-NS and EN-TiO_2−x_-NS. (**d**) UV-vis absorption spectra and (**e**) optical band gaps of the TiO_2_-NS, TiO_2−x_-NS and EN-TiO_2−x_-NS. (**f**) The scanning Kelvin probe maps of the TiO_2_-NS, TiO_2−x_-NS and EN-TiO_2−x_-NS.

The surface chemical states and defect structures of the samples before and after hydrogenation were characterized by XPS and electron paramagnetic resonance (EPR), with the results shown in Figure 3a–c. Figure 3a presents the high-resolution Ti 2p XPS spectra of pristine TiO_2_-NS, TiO_2−x_-NS, and EN-TiO_2−x_-NS. All three samples exhibit two characteristic peaks at 458.3 eV and 464.1 eV, corresponding to the spin–orbit splitting peaks of Ti 2p_3/2_ and Ti 2p_1/2_, respectively, indicating that titanium exists predominantly in the Ti^4+^ state. After the hydrogen reduction treatment, two new peaks appear simultaneously near 463.1 eV and 457.5 eV for both hydrogenated samples, which are assigned to the characteristic peaks of Ti^3+^ [44]. This indicates that the high-temperature hydrogen reduction successfully introduced Ti^3+^ species into the TiO_2_ lattice. The O 1s spectra of the samples could each be deconvoluted into two characteristic peaks, as shown in Figure 3b. The peak near 529.5 eV is attributed to lattice oxygen in TiO_2_, while the peak near 531.0 eV is attributed to oxygen vacancies [45]. Compared with the pristine TiO_2_-NS sample, the oxygen vacancy peak is relatively enhanced for both hydrogenated samples, indicating that the hydrogen reduction treatment introduced additional oxygen vacancy defects into the TiO_2_ lattice. Peak fitting of the Ti 2p spectra yields Ti^3+^ ratios of 21.7% and 21.0% for TiO_2−x_-NS and EN-TiO_2−x_-NS, respectively, while the O 1s spectra give oxygen vacancy proportions of approximately 36% and 35%. These comparable values confirm that the EDA pretreatment does not significantly affect defect introduction during hydrogenation (Tables S3 and S4).

Figure 3c presents the EPR spectra of the three samples. No obvious signal peak is detected for pristine TiO_2_-NS. In contrast, both hydrogenated samples, TiO_2−x_-NS and EN-TiO_2−x_-NS, exhibit a strong symmetric signal peak at g = 2.002, which is attributed to the paramagnetic center formed by oxygen vacancies trapping electrons [44,46]. This result is consistent with the detection of oxygen vacancy peaks in the XPS analysis, further confirming that the hydrogen reduction treatment successfully introduced oxygen vacancy defects into the TiO_2_ lattice. Comparing the two hydrogenated samples, the EPR signal intensity of EN-TiO_2−x_-NS is similar to that of TiO_2−x_-NS, indicating that the EDA reflux protection treatment did not affect the formation of oxygen vacancies during the hydrogenation process.

The optical absorption properties of the three samples were investigated by UV-Vis DRS. As shown in Figure 3d, pristine TiO_2_-NS exhibits strong absorption only in the ultraviolet region below 400 nm, characteristic of the intrinsic bandgap absorption of anatase TiO_2_. After hydrogen reduction, both TiO_2−x_-NS and EN-TiO_2−x_-NS display a new broad absorption band extending into the visible light region (400–800 nm), accompanied by a color change from white to light gray. This enhanced visible light absorption is attributed to the defect states (oxygen vacancies and Ti^3+^) introduced during hydrogenation, which create intermediate energy levels within the bandgap and enable the absorption of lower-energy photons. The corresponding band gap values derived from the Tauc plots are presented in Figure 3e. The calculated band gap of pristine TiO_2_-NS is 2.95 eV, while those of TiO_2−x_-NS and EN-TiO_2−x_-NS are 2.62 eV and 2.55 eV, respectively. To gain theoretical insight into the effect of oxygen vacancies on the electronic structure of the anatase TiO_2_ (001) surface, DFT band structure calculations were performed on pristine and oxygen-vacancy-containing (001) slabs. As shown in Figure S8, the calculated bandgap of the pristine (001) surface is 2.831 eV. Upon introducing an oxygen vacancy, the bandgap decreases significantly to 2.033 eV. This defect-induced bandgap narrowing is fully consistent with the experimentally observed trend. The higher absorption intensity of EN-TiO_2−x_-NS in the visible region may be attributed to its preserved nanosheet morphology, while other factors such as facet-dependent defect distribution and surface electronic effects may also contribute. The computational results therefore corroborate the experimental finding that oxygen vacancies and the accompanying Ti^3+^ species modify the electronic structure of the (001) surface, leading to enhanced visible light absorption. The band gap narrowing observed for both hydrogenated samples is consistent with the defect states identified by XPS and EPR, and the comparable degree of narrowing further confirms that the EDA reflux protection strategy does not adversely affect defect introduction during hydrogenation. Such combined analysis of XPS with structural and optical properties has been emphasized in previous studies on defect-engineered TiO_2_ photocatalysts [47,48]. However, given that the two hydrogenated samples possess comparable defect levels yet display markedly different photocatalytic activities, the enhanced performance of EN-TiO_2−x_-NS cannot be attributed to band gap narrowing alone. As evidenced by XRD and TEM, the preserved nanosheet morphology and (001) facets of EN-TiO_2−x_-NS likely enable more effective utilization of the introduced defects, a point that is further examined by the following SKP, EIS, and photocurrent measurements.

The surface work functions of the samples before and after hydrogenation were measured by scanning Kelvin probe (SKP), with the results shown in Figure 3f. The work function represents the minimum energy required to excite an electron from the Fermi level to the vacuum level, and serves as an important parameter reflecting the surface electronic state of a material. The pristine TiO_2_-NS sample exhibits a work function of 5.67 eV. After hydrogen reduction, the two hydrogenated samples show contrasting changes: TiO_2−x_-NS displays an increased work function of 5.84 eV, whereas EN-TiO_2−x_-NS shows a decreased work function of 5.51 eV. This divergence is notable given that XPS and EPR confirm comparable defect concentrations in both samples, indicating that factors beyond defect population govern the work function variation. The higher work function of TiO_2−x_-NS can be understood from its structural degradation. As revealed by XRD, SEM, and TEM, this sample underwent morphological collapse from nanosheets to nanoparticles, accompanied by the loss of (001) facets and the exposure of predominantly (101) facets. It has been established that the (001) facet of anatase TiO_2_ possesses a lower work function than the (101) facet due to the higher density of undercoordinated surface Ti atoms. Therefore, the facet reconstruction from (001) to (101) leads to an increase in work function, overriding any reduction that might be expected from defect introduction. In contrast, EN-TiO_2−x_-NS retains the nanosheet morphology and (001) facet exposure. The lower work function of EN-TiO_2−x_-NS implies a reduced energy barrier for electron transfer from the catalyst surface to adsorbed Cr(VI) species. This is also corroborated by the XPS and EPR results, confirming that the surface electronic properties are a key factor contributing to the excellent photocatalytic performance of EN-TiO_2−x_-NS. Overall, the SKP results demonstrate that the work function is governed not only by defect introduction but also, more critically, by the surface facet characteristics, reinforcing the conclusion that morphology preservation is essential for achieving efficient charge transfer and superior photocatalytic performance.

**Figure 4:**
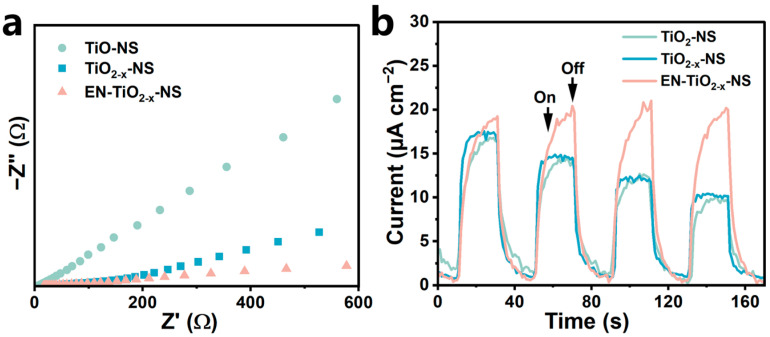
Electrochemical impedance spectroscopy (**a**) and transient photocurrent responses (**b**) of the TiO_2_-NS, TiO_2−x_-NS and EN-TiO_2−x_-NS.

The EIS and transient photocurrent response results are shown in Figure 4. Figure 4a presents the electrochemical impedance spectra of the three samples. As can be seen, the pristine TiO_2_-NS exhibits the highest interfacial charge transfer resistance. The directly hydrogenated TiO_2−x_-NS sample shows a lower resistance, indicating that the hydrogen reduction treatment reduces the interfacial charge transfer resistance to a certain extent. In contrast, the EN-TiO_2−x_-NS sample subjected to EDA reflux protection prior to hydrogenation possesses the lowest interfacial charge transfer resistance, demonstrating that it has the most favorable structure for the migration of photogenerated charge carriers. Figure 4b presents the transient photocurrent response curves of the three samples. As can be seen from the figure, the photocurrent densities of pristine TiO_2_-NS and the directly hydrogenated TiO_2−x_-NS sample are relatively close, whereas the photocurrent density of the EN-TiO_2−x_-NS sample is significantly higher than those of the former two. The photocurrent response intensity reflects the ability of photogenerated electrons to migrate from the interior of the catalyst to the surface and participate in reactions; a higher photocurrent indicates a higher separation efficiency of photogenerated electron-hole pairs. Based on the EIS and photocurrent test results, it can be seen that EN-TiO_2−x_-NS possesses the lowest interfacial charge transfer resistance and the highest photogenerated carrier separation efficiency. This can be attributed to a combination of factors. First, the EDA reflux treatment effectively preserves the nanosheet morphology and crystal surface structure at high temperature. Second, the hydrogen reduction generates oxygen vacancies and changes in Ti valence states, creating new defect states within the forbidden band that greatly reduce the energy barrier during charge transport; meanwhile, the modified surface work function facilitates electron movement. It is worth noting that although the directly hydrogenated TiO_2−x_-NS sample also contains certain surface defects, the changes in its morphology and crystal structure weaken the enhancement of charge separation efficiency. While the impedance of this material is reduced, its photocurrent does not show an obvious increasing trend. In contrast, the EN-TiO_2−x_-NS sample exhibits excellent charge separation and therefore performs well in subsequent photocatalytic experiments.

### 3.2. Evaluation of Photocatalytic Cr(VI) Reduction Performance

**Figure 5:**
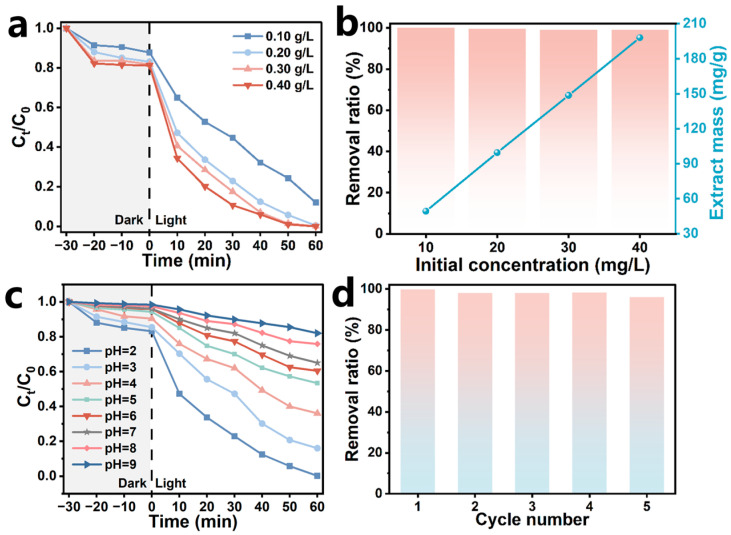
(**a**) Effect of photocatalyst dosage on Cr (VI) removal over TiO_2_-NS. (**b**) Effect of initial Cr (VI) concentration on Cr (VI) removal over TiO_2_-NS. (**c**) Effect of initial pH on Cr (VI) removal over TiO_2_-NS. (**d**) Cycling stability test of TiO_2_-NS.

To determine the optimal reaction conditions, TiO_2_-NS was used as a model catalyst, and the effects of catalyst dosage, initial Cr(VI) concentration, and initial pH on the photocatalytic reduction efficiency were systematically investigated. The stability of the catalyst was also evaluated through recycling experiments. The results are shown in Figure 5. Figure 5a shows the effect of catalyst dosage on Cr(VI) removal efficiency. Under an initial Cr(VI) concentration of 10 mg/L and pH = 2, the Cr(VI) removal efficiency gradually increased as the TiO_2_-NS dosage was raised from 0.1 g/L to 0.4 g/L. At a dosage of 0.2 g/L, the Cr(VI) removal rate exceeded 99% within 60 min. Further increasing the dosage to 0.3 g/L and 0.4 g/L resulted in only marginal improvement with no significant difference. Considering both catalytic efficiency and material consumption, 0.2 g/L was selected as the catalyst dosage for subsequent experiments. The reaction rate constants under the aforementioned light conditions are shown in the Supporting Information (Figure S9). Figure 5b presents the influence of initial Cr(VI) concentration on removal efficiency and removal capacity. Under a catalyst dosage of 0.2 g/L and pH = 2, the photocatalytic reduction performance was examined at initial Cr(VI) concentrations of 10, 20, 30, and 40 mg/L. The results show that the final Cr(VI) removal rate exceeded 99% at all concentrations after light irradiation. As the initial concentration increased, the removal capacity per unit mass of catalyst gradually increased. At an initial concentration of 40 mg/L, the maximum removal capacity of TiO_2_-NS reached 198.0 mg·g^−1^, demonstrating good treatment capability. Figure 5c illustrates the effect of initial pH on Cr(VI) removal efficiency. With an initial Cr(VI) concentration of 10 mg/L and a catalyst dosage of 0.2 g/L, the photocatalytic reduction efficiency was studied at pH values of 2–9. The results indicate that the Cr(VI) removal rate decreased gradually as the pH increased. At pH = 2, the removal rate exceeded 99% within 60 min, whereas at pH = 9, the removal rate dropped significantly. This phenomenon can be attributed to the pH-dependent speciation of Cr(VI) and the surface charge state of TiO_2_. Under strongly acidic conditions, Cr(VI) exists predominantly as HCrO_4_^−^, and the TiO_2_ surface is positively charged due to protonation of surface hydroxyl groups. This electrostatic attraction facilitates the adsorption of Cr(VI) anions onto the catalyst and promotes the subsequent reduction to Cr(III). As the pH increases, the TiO_2_ surface charge becomes less positive, reducing the electrostatic affinity for Cr(VI). Under alkaline conditions (pH > 7), Cr(VI) is mainly present as CrO_4_^2−^, and the TiO_2_ surface becomes negatively charged, leading to electrostatic repulsion that severely suppresses adsorption and photoreduction. Additionally, Cr(III) species may precipitate as Cr(OH)_3_ on the catalyst surface under alkaline conditions, blocking active sites and further deteriorating the performance [49,50]. Figure 5d shows the cycling stability test results of the TiO_2_-NS sample for the photocatalytic reduction in Cr(VI). After five consecutive cycles, the Cr(VI) removal rate of TiO_2_-NS remained above 96%, indicating that the catalyst possesses good stability and reusability.

**Figure 6:**
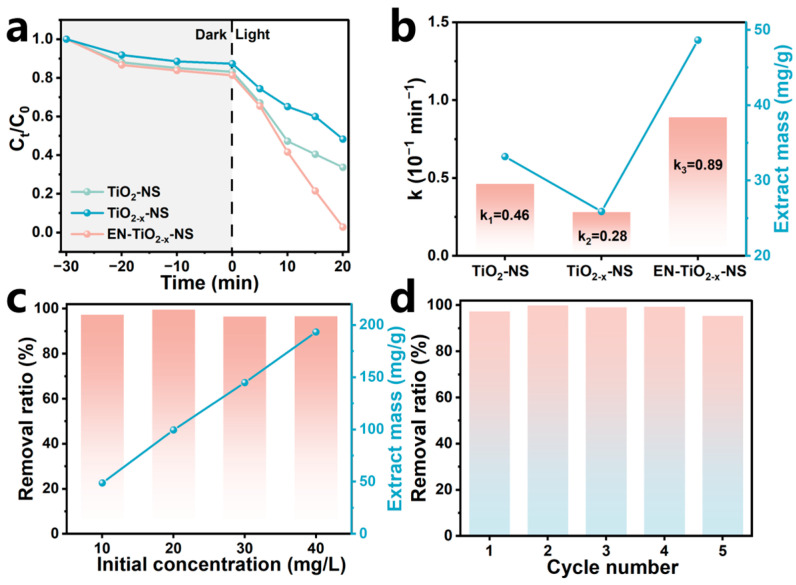
(**a**) Photocatalytic removal kinetics of Cr(VI) by various photocatalysts. (**b**) Reaction rate constant (k) under light and extract mass for various photocatalysts. (**c**) Effect of initial Cr (VI) concentration on Cr (VI) removal over EN-TiO_2−x_-NS. (**d**) Cycling stability test of EN-TiO_2−x_-NS.

Based on the above optimized conditions (catalyst dosage 0.2 g/L, pH = 2), the photocatalytic reduction performance of pristine TiO_2_-NS, TiO_2−x_-NS, and EN-TiO_2−x_-NS for Cr(VI) was further evaluated, with the results shown in Figure 6. Figure 6a displays the photocatalytic reduction kinetic curves of Cr(VI) over the three samples. Before light irradiation, the suspension was stirred in the dark for 30 min to reach adsorption–desorption equilibrium. Preliminary time-dependent dark adsorption tests confirmed that equilibrium was achieved within 10 min, and the subsequent 30 min dark stirring was sufficient for the samples to reach adsorption–desorption equilibrium (Figure S10). It can be seen that during the dark reaction stage, TiO_2_-NS and EN-TiO_2−x_-NS exhibited comparable adsorption capacities for Cr(VI), while TiO_2−x_-NS showed the poorest adsorption capacity. This may be due to the loss of surface-active sites caused by the morphological and crystal facet transformation of the directly hydrogenated sample.

Upon light irradiation, the removal rate of Cr(VI) by EN-TiO_2−x_-NS reached 97.3% within 20 min of visible light exposure, whereas the removal rates of pristine TiO_2_-NS and directly hydrogenated TiO_2−x_-NS were 66.3% and only 51.7%, respectively. This indicates that the strategy combining EDA reflux protection with hydrogen reduction can significantly enhance the photocatalytic reduction ability of TiO_2_. Given that TiO_2−x_-NS and EN-TiO_2−x_-NS possess comparable defect concentrations, the significant difference in their photocatalytic activities suggests that morphology preservation may play an important role. The retained nanosheet structure and (001) facets of EN-TiO_2−x_-NS likely facilitate more effective utilization of the defects in photocatalysis, while the directly hydrogenated product without EDA protection suffered a drastic decrease in photocatalytic activity, even falling below that of pristine TiO_2_-NS, due to changes in morphology and crystal structure.

The photocatalytic reduction processes were fitted by a pseudo-first-order kinetic model (Figure 6b), yielding apparent rate constants of 0.046 min^−1^, 0.028 min^−1^, and 0.089 min^−1^ for TiO_2_-NS, TiO_2−x_-NS, and EN-TiO_2−x_-NS, respectively. This model is widely applied to photocatalytic Cr(VI) reduction under constant catalyst loading and light intensity, where the reaction rate is proportional to the Cr(VI) concentration. The rate constant of EN-TiO_2−x_-NS was 1.93 times that of pristine TiO_2_-NS and 3.17 times that of TiO_2−x_-NS. The significantly higher rate constant of EN-TiO_2−x_-NS can be attributed to the combined effect of its preserved morphology and the introduced defects. The retained nanosheet structure and exposed (001) facets provide abundant surface-active sites for Cr(VI) reduction and facilitate charge transfer, enabling the introduced oxygen vacancies and Ti^3+^ to contribute more effectively to the reduction kinetics. In contrast, although TiO_2−x_-NS possesses a comparable defect concentration, it loses this structural advantage due to the collapse of the nanosheet morphology during unprotected hydrogenation. Consequently, the defects in TiO_2−x_-NS cannot be fully utilized, resulting in a rate constant even lower than that of pristine TiO_2_-NS. This comparison demonstrates that defect introduction provides the necessary driving force for enhanced photocatalytic activity, while morphology preservation ensures that this potential can be fully realized. Under the same light conditions, the maximum Cr(VI) removal capacities of the three materials also differed: EN-TiO_2−x_-NS achieved 48.6 mg·g^−1^, while TiO_2_-NS and TiO_2−x_-NS reached 33.2 mg·g^−1^ and 25.9 mg·g^−1^, respectively. The higher removal capacity of EN-TiO_2−x_-NS further confirms its superior photocatalytic reduction performance. The reaction rate constants under the aforementioned light conditions are shown in the Supporting Information (Figure S11). A comparison with representative Ti-based photocatalysts in the reported literature (Table S5) shows that EN-TiO_2−x_-NS achieves comparable or superior performance in Cr(VI) reduction, further demonstrating its excellent photocatalytic properties [50,51,52,53,54,55,56,57].

To further optimize the photocatalytic performance, we varied the reduction temperature (400–600 °C) and EDA reflux time (2–72 h) (Figures S12 and S13). The optimal performance was achieved at 500 °C with 48 h of reflux. Lower reduction temperatures (400 °C) yielded limited defect generation, whereas excessive temperature (600 °C) led to structural collapse and loss of (001) facets, diminishing the photocatalytic performance despite higher defect concentrations. Regarding the EDA protection, insufficient reflux time (2 h) failed to establish a complete protection, rendering the nanosheets susceptible to collapse during hydrogen reduction, while extending reflux to 72 h showed no additional benefit, suggesting saturated surface coverage. These results confirm that the enhanced activity arises from the synergistic effect of morphology preservation and defect engineering, and that an optimal balance between the two is critical for maximizing performance. Under the optimal conditions, the photocatalytic reduction performance of EN-TiO_2−x_-NS at different initial Cr(VI) concentrations and its cycling stability were further investigated. Figure 6c shows the effect of initial Cr(VI) concentration on removal efficiency and removal capacity. With an EN-TiO_2−x_-NS dosage of 0.2 g/L and pH = 2, the photocatalytic reduction performance was tested at initial Cr(VI) concentrations of 10, 20, 30, and 40 mg/L. The results indicate that within the tested concentration range, the final Cr(VI) removal rate exceeded 95% at all concentrations after light irradiation. As the initial concentration increased, the removal capacity per unit mass of catalyst gradually increased. At an initial concentration of 40 mg/L, the maximum removal capacity of EN-TiO_2−x_-NS reached 193.3 mg·g^−1^, demonstrating good treatment capability. Figure 6d presents the cycling stability test results of EN-TiO_2−x_-NS for the photocatalytic reduction in Cr(VI). After each cycle, the catalyst was recovered by centrifugation, washed with deionized water, and redispersed in a freshly prepared Cr(VI) solution for the next photocatalytic reaction. It can be seen from the figure that after five consecutive cycles, the Cr(VI) removal rate of EN-TiO_2−x_-NS remained above 95%, indicating that the catalyst possesses good stability.

To further understand the slight activity decline observed in the fifth cycle, the spent EN-TiO_2−x_-NS catalyst was characterized by XRD, TEM, XPS, and EPR. XRD and TEM analyses confirm that the anatase phase and nanosheet morphology are well preserved after five consecutive cycles, demonstrating the excellent structural stability of the catalyst (Figure S14a,b). Meanwhile, EPR analysis shows a slight decrease in the signal intensity at g = 2.002 compared with the fresh catalyst, suggesting partial re-oxidation of surface oxygen vacancies during repeated use (Figure S14c). The XPS Cr 2p spectrum of the spent catalyst can be deconvoluted into four peaks at 575.06, 578.92, 586.32, and 588.87 eV (Figure S14d). The peaks at 575.06 and 586.32 eV are characteristic of Cr(III), while those at 578.92 and 588.87 eV correspond to Cr(VI) [58]. The coexistence of Cr(III) and Cr(VI) confirms that a portion of the reduced Cr(III) remains adsorbed on the catalyst surface after the reaction, which may partially block the active sites and contribute to the gradual decline in activity. These combined surface-level changes, namely chromium accumulation and partial defect loss, rather than structural degradation, are likely responsible for the minor activity decline.

To identify the reactive species responsible for the photocatalytic Cr(VI) reduction, radical scavenging experiments were performed over EN-TiO_2−x_-NS. AgNO_3_ (1 mM), p-benzoquinone (1 mM), and isopropanol (10 mM) were employed as scavengers for photogenerated electrons (e^−^), superoxide radicals (•O_2_^−^), and hydroxyl radicals (•OH) [56], respectively. As shown in Figure S15, the addition of AgNO_3_ almost completely suppressed the Cr(VI) reduction, confirming that photogenerated electrons are the dominant reactive species driving the reduction process. The addition of p-benzoquinone resulted in a slight decrease in removal efficiency (from 97.3% to 88%), indicating that •O_2_^−^ radicals, generated from the reaction of electrons with dissolved O_2_, also contribute to the reduction, although to a lesser extent. In contrast, the addition of IPA led to an enhanced reduction rate, which can be attributed to the consumption of photogenerated holes by IPA, thereby suppressing electron-hole recombination and facilitating more efficient electron transfer to Cr(VI). This also confirms that •OH radicals are not directly involved in the Cr(VI) reduction process. These results collectively demonstrate that photogenerated electrons are the primary active species, with •O_2_^−^ playing a secondary role, in the photocatalytic Cr(VI) reduction over EN-TiO_2−x_-NS.

## 4. Conclusions

This work proposes an EDA reflux protection strategy combined with hydrogen reduction to prevent the morphological collapse and facet reconstruction of anatase TiO_2_ nanosheets during high-temperature hydrogenation, aiming to preserve the highly active morphology while efficiently introducing defects. The protected EN-TiO_2−x_-NS fully retains the original nanosheet morphology and (001) facets, whereas the unprotected TiO_2−x_-NS transforms into irregular nanoparticles. XPS and EPR confirm that comparable levels of Ti^3+^ and oxygen vacancies are introduced into EN-TiO_2−x_-NS. Consequently, its band gap narrows from 2.95 eV to 2.55 eV, visible light absorption is enhanced, and charge separation efficiency is markedly improved. In photocatalytic Cr(VI) reduction, EN-TiO_2−x_-NS achieves a removal rate of 97.3% within 20 min, with rate constants 1.93 and 3.17 times those of pristine and directly hydrogenated samples, respectively, and exhibits excellent cycling stability. In contrast, the directly hydrogenated sample shows even poorer performance than the pristine one despite containing defects, highlighting the critical importance of preserving the active structure during defect engineering. This strategy successfully integrates morphological stabilization with efficient defect introduction, offering a new approach for designing TiO_2_-based photocatalysts with both wide spectral response and high activity.

## Data Availability

The original contributions presented in this study are included in the article/Supplementary Material. Further inquiries can be directed to the corresponding authors.

## Supplementary Materials

The following supporting information can be downloaded at: https://www.mdpi.com/article/10.3390/nano16130832/s1, Figure S1. (a,b) TEM and HRTEM images of TiO_2−x_-NS. Figure S2. Nitrogen adsorption/desorption isotherms of TiO_2_-NS. Figure S3. Nitrogen adsorption/desorption isotherms of TiO_2−x_-NS. Figure S4. Nitrogen adsorption/desorption isotherms of EN-TiO_2−x_-NS. Figure S5. FT-IR spectra of EN-TiO_2_-NS (Before reduction) and EN-TiO_2−x_-NS. Figure S6. XPS N 1s spectrum of EN-TiO_2_-NS (After EDA treated) intermediate before calcination. Figure S7. XPS survey spectra of the EN-TiO_2_-NS (Before reduction) and EN-TiO_2−x_-NS. Figure S8. Energy band structures of TiO_2_ (001) (a) and TiO_2−x_ (001) (b). Figure S9. Reaction rate constant (k) for Cr(VI) photoreduction by TiO_2_-NS at different catalyst dosages. Figure S10. Cr(VI) removal by EN-TiO_2−x_-EN under dark conditions. Figure S11. Reaction rate constants (k) of Cr(VI) photoreduction over different catalysts. Figure S12. Effect of hydrogen reduction temperature on the photocatalytic Cr(VI) reduction performance of EN-TiO_2−x_-NS. Figure S13. Effect of EDA reflux time on the photocatalytic Cr(VI) reduction performance of EN-TiO_2−x_-NS. Figure S14. Post-reaction characterization of the spent EN-TiO_2−x_-NS catalyst after five cycles. (a) XRD pattern, (b) TEM image, (c) EPR spectrum, and (d) XPS Cr 2p spectrum. Figure S15. Radical scavenging experiments for the photocatalytic Cr(VI) reduction over EN-TiO_2−x_-NS. Table S1. Comparison of different strategies for introducing oxygen vacancies. Table S2. The surface area (m^2^/g) and Pore Volume (cm^3^/g) of TiO_2_-NS, TiO_2−x_-NS and EN-TiO_2−x_-NS. Table S3. Proportions of O_L_ and O_V_ based on O 1s XPS spectra of TiO_2−x_-NS and EN-TiO_2−x_-NS. Table S4. Proportions of Ti^3+^ and Ti^4+^ based on Ti 2p XPS spectra of TiO_2−x_-NS and EN-TiO_2−x_-NS. Table S5. Comparison of the photocatalytic Cr(VI) reduction performance of EN-TiO_2−x_-NS with other Ti-based photocatalysts reported in the literature.
