# Supplementary material for: Oxygen-Vacancy-Rich TiO2 Nanosheets with High Stability for Efficient Photocatalytic Cr(VI) Reduction
Source: Nanomaterials (Basel). 2026 Jul 7;16(13):832. doi: 10.3390/nano16130832 (PMC13363494; doi:10.3390/nano16130832)
Supplement: Supplementary file 1 [file nanomaterials-16-00832-s001.zip › nanomaterials-4395823-supplementary.pdf]

Supplementary Information

Oxygen-Vacancy-Rich TiO_2 Nanosheets with High Stability for Efficient Photocatalytic Cr(VI) Reduction

Yingjie Jiang [1], Xiaoli Jia [1], Li Fang [1], Qin Zhang [1], Ruiting Li [1], Bingqian Zhao [1], Jiancong Liu [1,*] and Yaorui Li [2,*]

1 Key Laboratory of Functional Inorganic Material Chemistry, Ministry of Education of the People's Republic of China, Heilongjiang University, Harbin 150080, China

2 Heilongjiang Provincial Key Laboratory of Nuclear Chemical Engineering and Radiochemistry, College of Nuclear Science and Technology, Harbin Engineering University, Harbin 150001, China

* Correspondence: liujiancong@hlju.edu.cn or jiancong@gmail.com (J.L.); liyr@hrbeu.edu.cn (Y.L.)

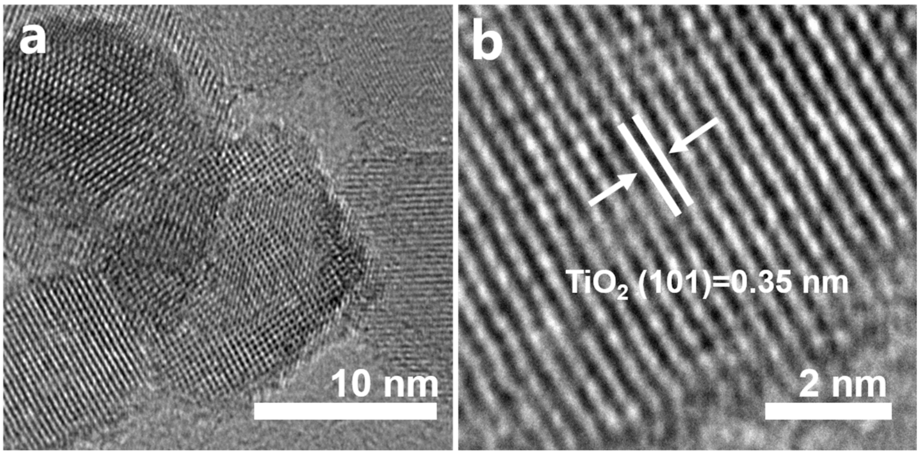

Figure S1. (a,b) TEM and HRTEM images of TiO_{2-x}-NS.

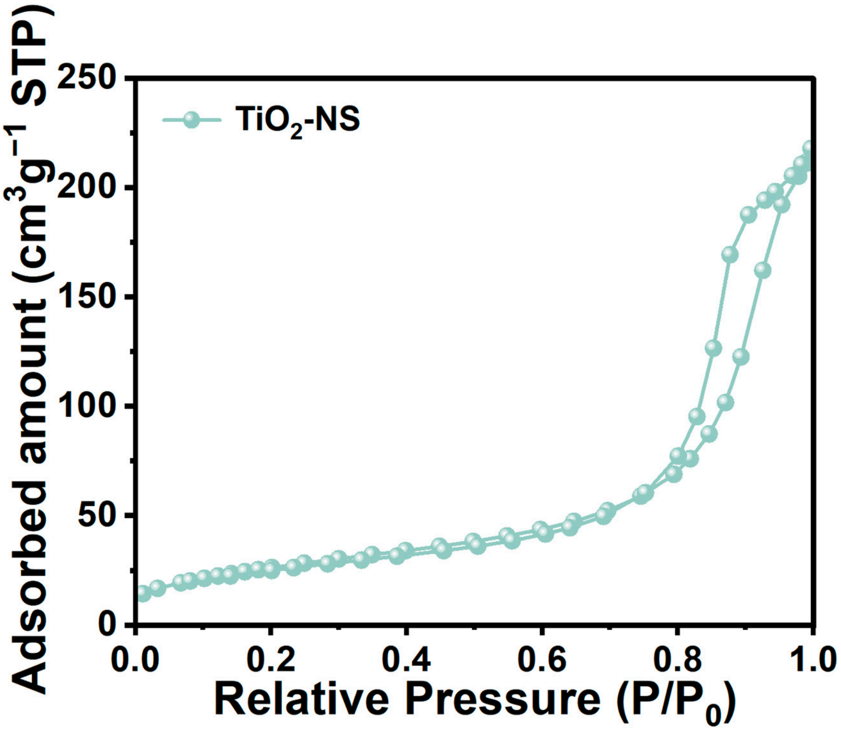

Figure S2. Nitrogen adsorption/desorption isotherms of TiO_2-NS.

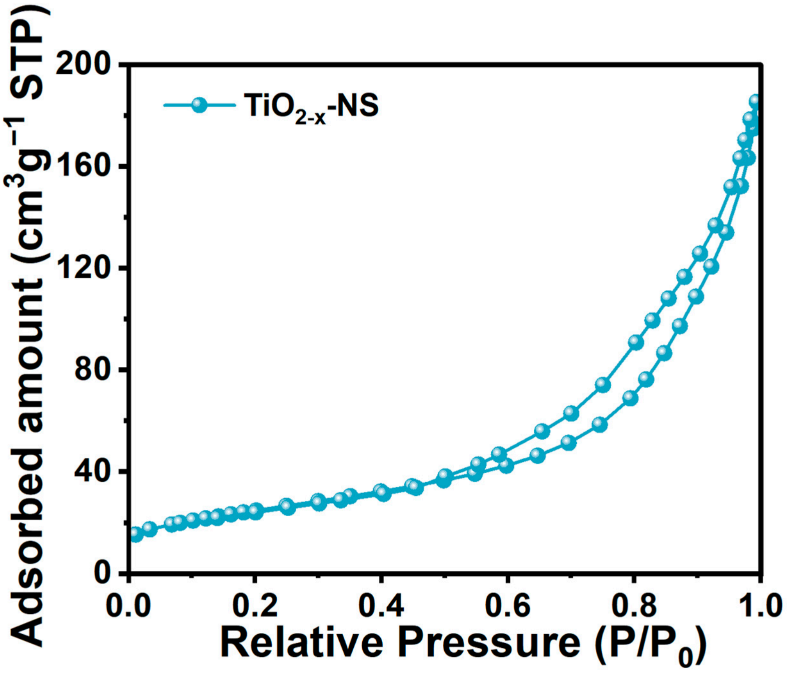

Figure S3. Nitrogen adsorption/desorption isotherms of TiO_{2-x}-NS.

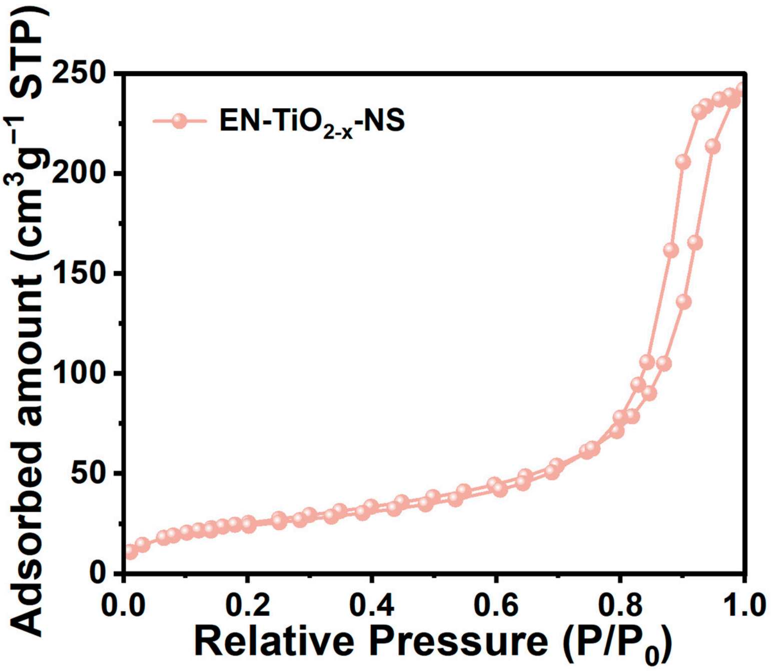

Figure S4. Nitrogen adsorption/desorption isotherms of EN-TiO_{2-x}-NS.

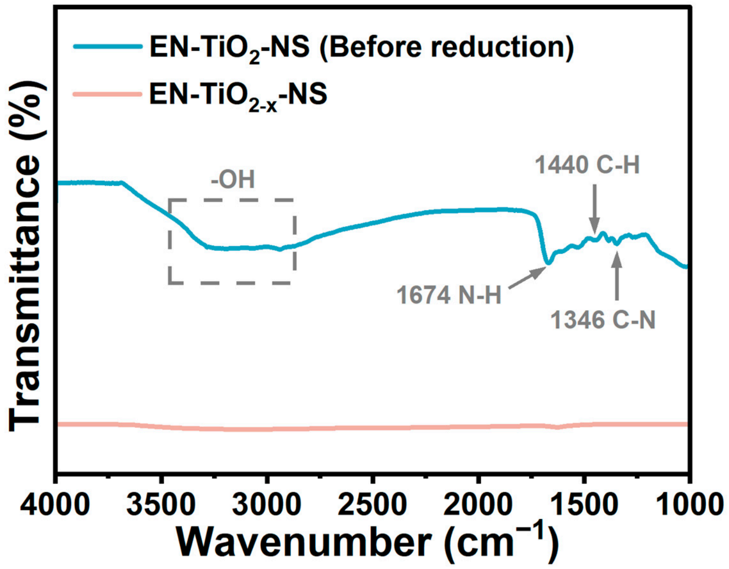

Figure S5. FT-IR spectra of EN-TiO_2-NS (Before reduction) and EN-TiO_{2-x}-NS.

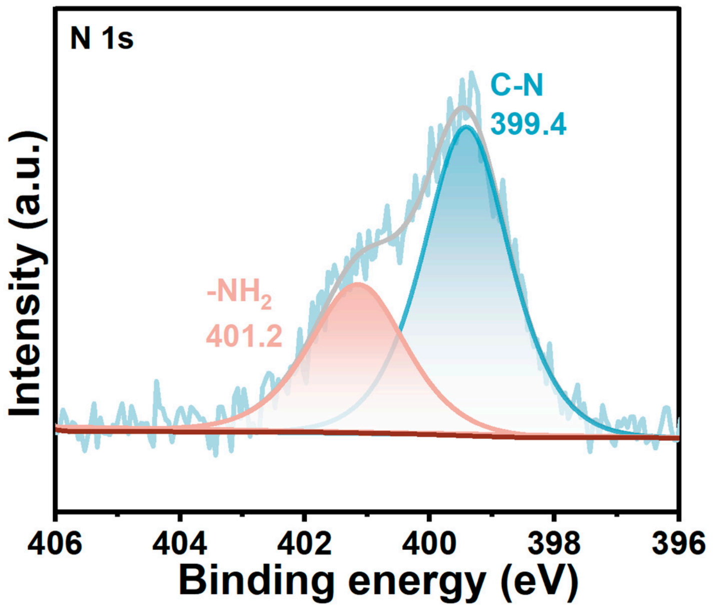

Figure S6. XPS N 1s spectrum of EN-TiO_2-NS (After EDA treated) intermediate before calcination.

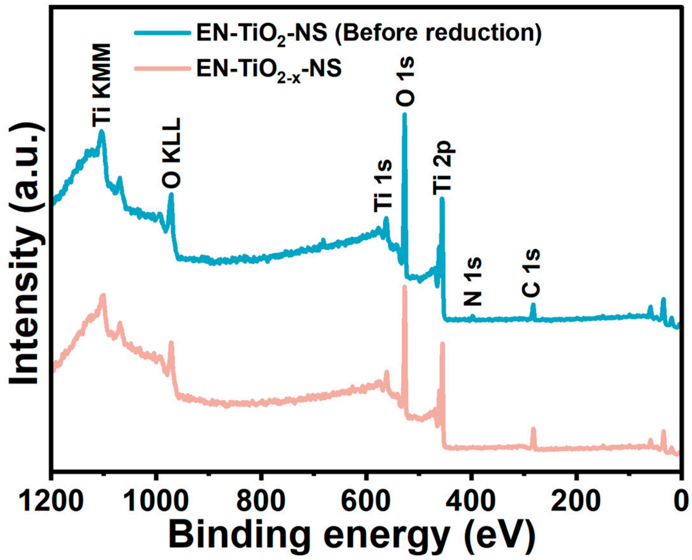

Figure S7. XPS survey spectra of the EN-TiO_2-NS (Before reduction) and EN-TiO_{2-x}-NS.

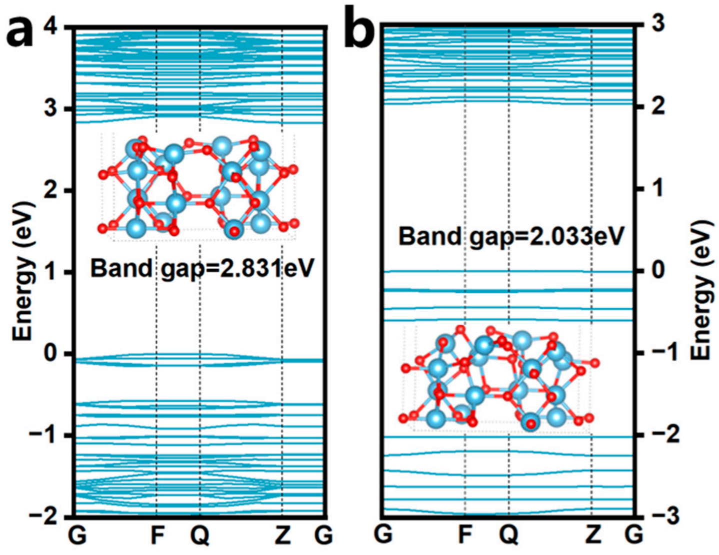

Figure S8. Energy band structures of TiO_2 (001) (**a**) and TiO_{2-x} (001) (**b**).

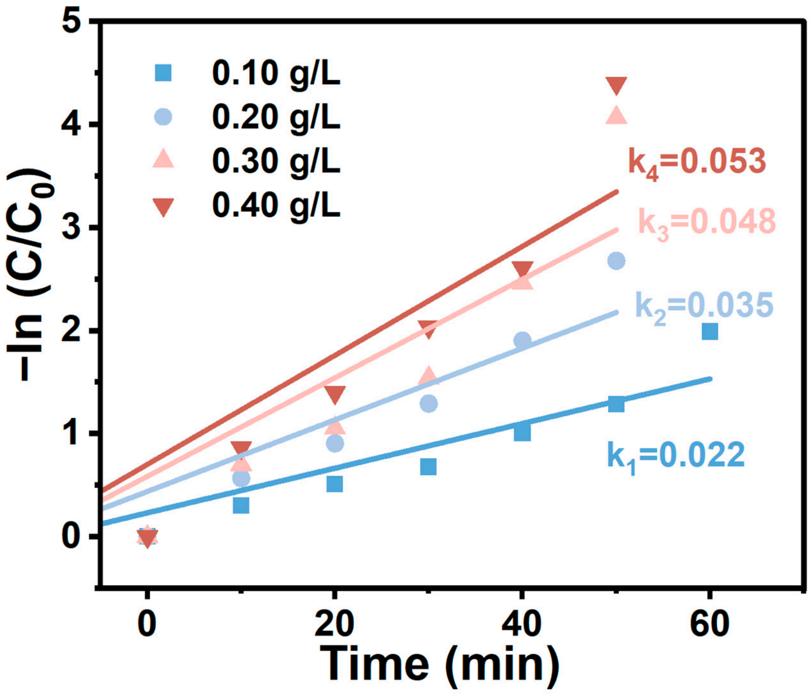

Figure S9. Reaction rate constant (k) for Cr(VI) photoreduction by TiO_2-NS at different catalyst dosages.

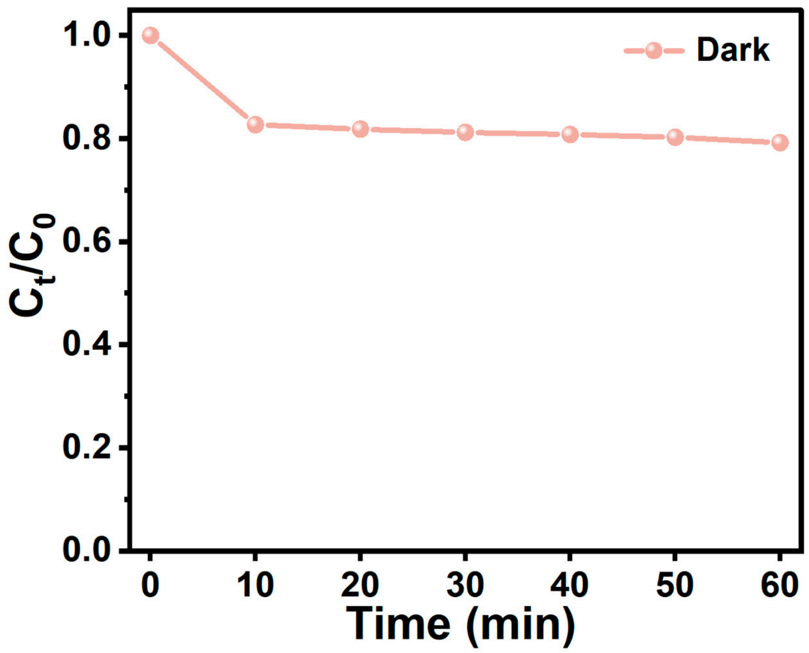

Figure S10. Cr(VI) removal by EN-TiO_{2-x}-EN under dark conditions.

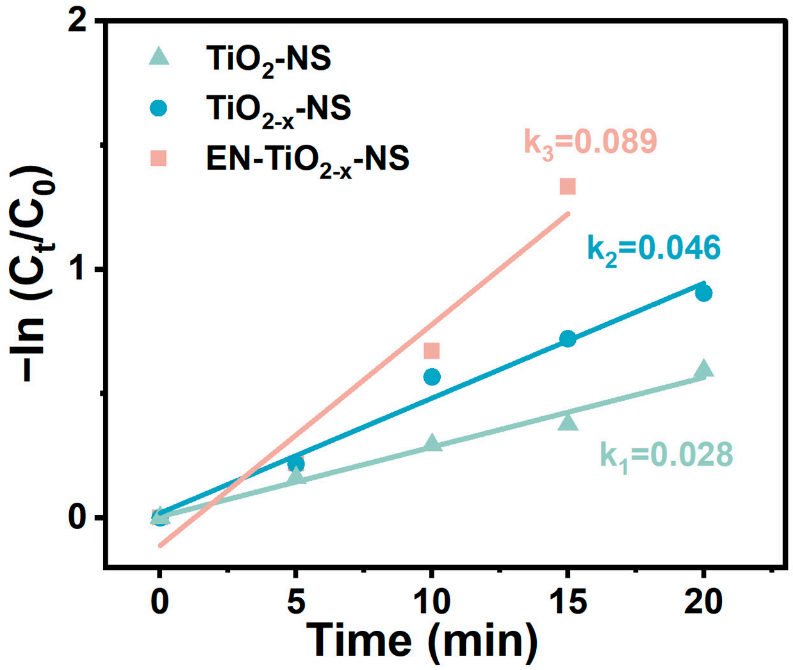

Figure S11. Reaction rate constants (k) of Cr(VI) photoreduction over different catalysts.

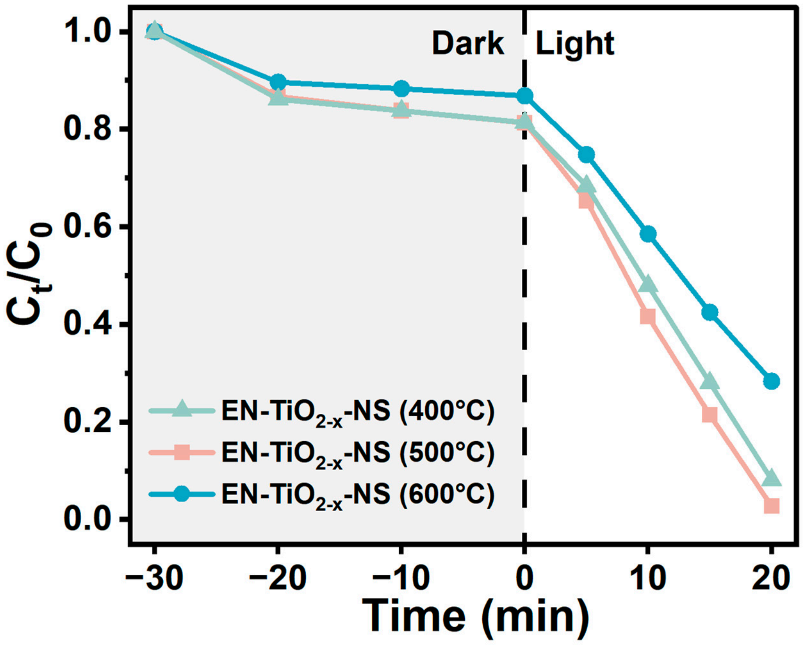

Figure S12. Effect of hydrogen reduction temperature on the photocatalytic Cr(VI) reduction performance of EN-TiO_{2-x}-NS.

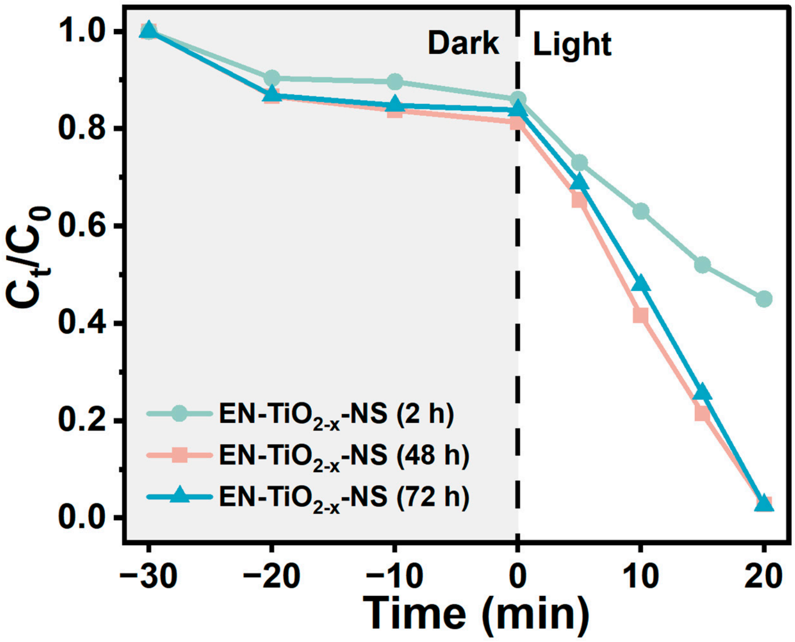

Figure S13. Effect of EDA reflux time on the photocatalytic Cr(VI) reduction performance of EN-TiO_{2-x}-NS.

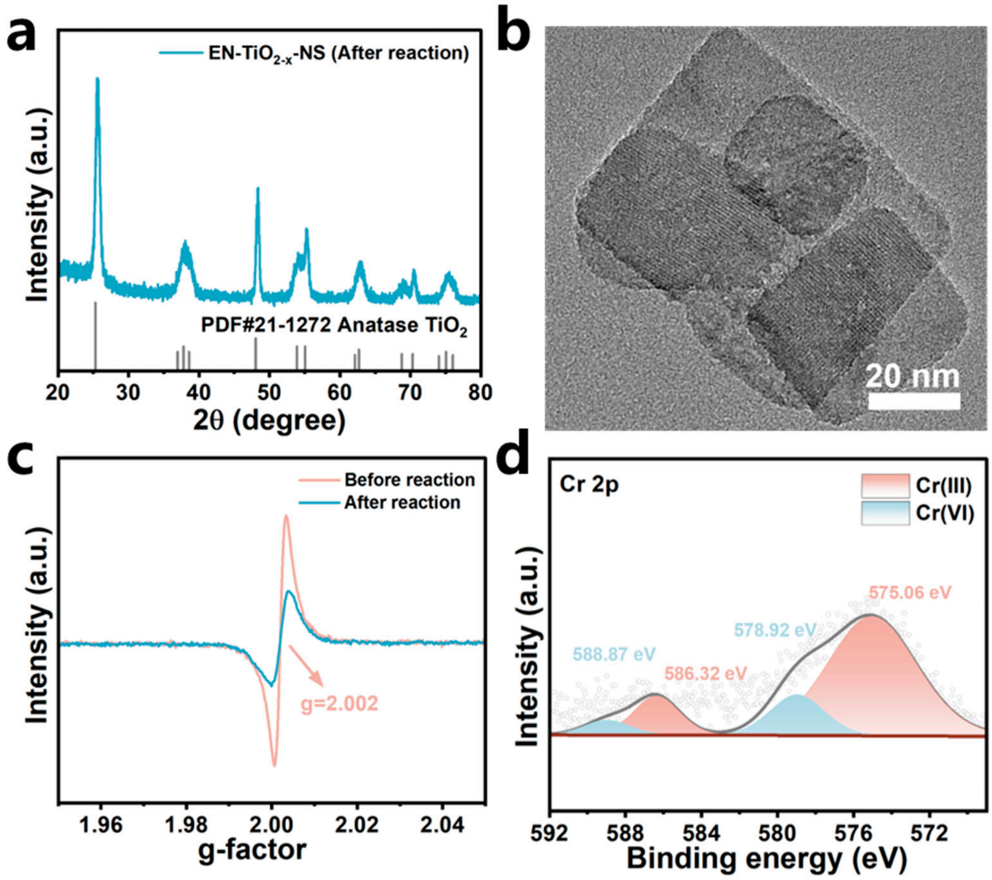

Figure S14. Post-reaction characterization of the spent EN-TiO_{2-x}-NS catalyst after five cycles. (**a**) XRD pattern, (**b**) TEM image, (**c**) EPR spectrum, and (**d**) XPS Cr 2p spectrum.

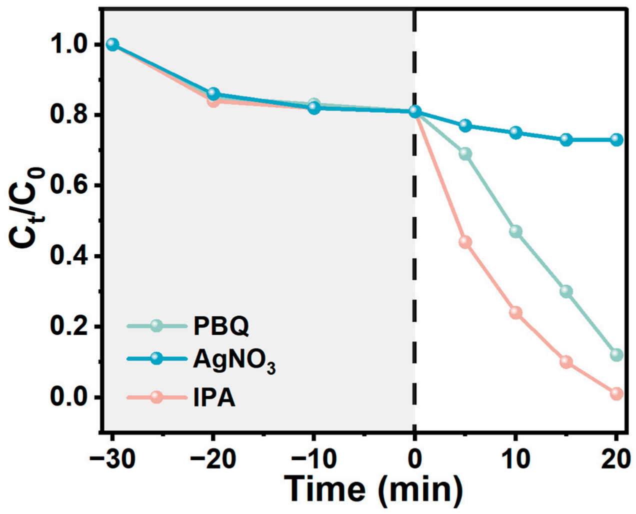

Figure S15. Radical scavenging experiments for the photocatalytic Cr(VI) reduction over EN-TiO_{2-x}-NS.

Table S1. Comparison of different strategies for introducing oxygen vacancies.

Method	Characteristics	Limitations	Ref.
Metal element doping	Convenience; synergistic electronic modulation	Limited defect concentration; foreign element introduction	[29,30]
Heterojunction induction	Interfacial effects to promote defect formation	Defects confined to interfacial region	[31]
Chemical reduction (e.g., $NaBH_4$)	Mild and simple operation	Low concentration of surface defects	[32]
High-temperature hydrogen reduction	High bulk defect concentration; marked bandgap narrowing	Structural collapse and material sintering	[33,34]
EDA reflux-assisted hydrogenation	Defect introduction while preserving morphology		This work

Table S2. The surface area (m^2/g) and Pore Volume (cm^3/g) of TiO_2-NS, TiO_{2-x}-NS and EN-TiO_{2-x}-NS.

Samples	Surface area (m^2/g)	Pore Volume (cm^3/g)
TiO_2-NS	98	0.27
TiO_{2-x}-NS	90	0.22
EN-TiO_{2-x}-NS	96	0.30

Table S3. Proportions of O_L and O_V based on O 1s XPS spectra of TiO_{2-x}-NS and EN-TiO_{2-x}-NS.

Samples	O_L (%)	O_V (%)
TiO_{2-x}-NS	64	36
EN-TiO_{2-x}-NS	65	35

Table S4. Proportions of Ti^{3+} and Ti^{4+} based on Ti 2p XPS spectra of TiO_{2-x}-NS and EN-TiO_{2-x}-NS.

Samples	Ti^{3+} (%)	Ti^{4+} (%)
TiO_{2-x}-NS	21.7	78.3
EN-TiO_{2-x}-NS	21	79

Table S5. Comparison of the photocatalytic Cr(VI) reduction performance of EN-TiO_{2-x}-NS with other Ti-based photocatalysts reported in the literature.

Materials	Irradiation	Removal rate (%)	Time (min)	Cr(VI) concentration	m/V	pH	k (min^{-1})	Ref.
EN-TiO_{2-x}-NS	300 W xenon lamp	97.3	20	10 mg/L	0.2	2	0.089	This work
MT-2	300 W xenon lamp	100	60	5 mg/L	0.3	2	0.1059	[51]
TiO_2-$Cu_2(OH)PO_4$	250 W high-pressure mercury lamp	100	90	10 mg/L	1	3	-	[52]
MIL-125(Ti)	Four 16 W UV-C (200 nm)	99.4	120	10 mg/L	1	2	-	[53]
NSC-TiO_2	500 W xenon lamp	100	50	10 mg/L	0.2	1.5	-	[54]
NH_2- MIL-125(Ti)	500 W xenon lamp	97	60	48 mg/L	0.4	2.1		[55]
UiO-66-NH_2@HDU-25	＞420 nm	99	30	15	0.2	3	0.138	[50]
BTD-OH-COF	＞420 nm	99.9	120	15	0.1	3	0.035	[56]
Ag_2S/BiSI	365 nm	88.7	40	20	-	-	0.0388	[57]
